# FTO gene expression in diet-induced obesity is downregulated by *Solanum* fruit supplementation

**DOI:** 10.1515/biol-2022-0067

**Published:** 2022-06-15

**Authors:** Edeke Affiong Asuquo, Okwesilieze Fred Chiletugo Nwodo, Anosike Chioma Assumpta, Uchendu Nene Orizu, Okoro Nkwachukwu Oziamara, Odiba Arome Solomon

**Affiliations:** Department of Biochemistry, Faculty of Biological Sciences, University of Nigeria, 410001, Nsukka, Enugu State, Nigeria; Department of Pharmaceutical and Medicinal Chemistry, Faculty of Pharmaceutical Sciences, University of Nigeria, 410001, Nsukka, Enugu State, Nigeria; Department of Molecular Genetics and Biotechnology, Faculty of Biological Sciences, University of Nigeria, 410001, Nsukka, Enugu State, Nigeria

**Keywords:** adipose tissues, fat mass and obesity-associated gene, hypothalamus, obesity, *Solanum aethiopicum*, *Solanum melongena*

## Abstract

The Fat Mass and Obesity-associated (FTO) gene has been shown to play an important role in developing obesity, manifesting in traits such as increased body mass index, increased waist-to-hip ratio, and the distribution of adipose tissues, which increases the susceptibility to various metabolic syndromes. In this study, we evaluated the impact of fruit-based diets of *Solanum melongena* (SMF) and *Solanum aethiopicum* fruits (SAF) on the FTO gene expression levels in a high-fat diet (HFD)-induced obese animals. Our results showed that the mRNA level of the FTO gene was downregulated in the hypothalamus, and white and brown adipose tissue following three and six weeks of treatment with SMF- and SAF-based diets in the HFD-induced obese animals. Additionally, the *Solanum* fruit supplementation exhibited a curative effect on obesity-associated abrasions on the white adipose tissue (WAT), hypothalamus, and liver. Our findings collectively suggest the anti-obesity potential of SMF and SAF via the downregulation of the FTO gene.

## 1 Introduction

According to the World Health Organization, obesity has nearly tripled worldwide since 1975 [1]. Data in 2016 showed that over 1.9 billion adults aged 18 years or older were overweight, of which over 650 million were obese, while over 340 million individuals aged 5–9 years were either obese or overweight [1]. Several factors, including diet and lifestyle and an individual’s genetic composition, could contribute to obesity [2]. The fat mass and obesity-associated (FTO) gene is one of the genetic factors known to contribute to polygenic obesity (obesity caused by a combination of genetic variants) and is involved in body weight and adiposity regulation. Fat mass, leptin levels, waist-to-hip ratio, increased food intake, and decreased satiety have all been linked to FTO gene mutations, upregulation, and single nucleotide polymorphisms [3,4,5]. In a previous study, Wahlen and colleagues reported an increased level of subcutaneous adipose tissue FTO mRNA in obese individuals compared to non-obese subjects [6]. Expression of the FTO gene is ubiquitous in the cell nucleus of almost all human tissues, and its levels are high in the adipose tissues as well as in the arcuate nucleus of the hypothalamus, which is known to play a major role in the control of energy homeostasis and eating behavior [4,7]. The hypothalamus, brown adipose tissue (BAT), white adipose tissue (WAT), and liver are all connected. It is important to note that obesity is related to energy status, and the central nervous system (CNS) orchestrates the energy balance [8,9], with the hypothalamus playing a critical role. The majority of peripheral cues, such as sensory and nutritional signals and humoral elements, reach the hypothalamus to provide information about the different energy conditions [10]. Most significantly, hypothalamic circuits control BAT thermogenesis and WAT browning. The preoptic region (POA), triggered by cold and prostaglandin E2, gets peripheral information about external temperature and mediates the febrile response. Through projections to the rostral raphe nucleus, the POA projects to other hypothalamic nuclei, including the ventromedial nucleus (VN) of the hypothalamus, activating the BAT sympathetic flow (rRPa) [11,12]. Numerous peripheral signals converge in the VN to regulate thermogenesis. For example, the bone morphogenic protein 8 receptor (BMP8R) inhibits AMPK in the VN, hence increasing thermogenesis in brown and WATs [10,11,12]. The liver plays a very critical role in maintaining energy homeostasis. For example, liver X receptors (LXRs), which are highly expressed in the liver, play a critical role in maintaining cholesterol, fatty acid, and glucose homeostasis [13,14,15,16]. Additionally, previous research has demonstrated that genetic deletion of LXRs results in the browning of WAT via stimulation of thyrotropin-releasing hormone in the hypothalamic paraventricular nucleus [17].

Obesity has been induced in laboratory animals by various methods [18,19,20,21,22]. Identifying an animal model capable of mimicking human biological processes is critical for comprehending human diseases such as obesity and its comorbidities. Based on the physiological and genetic similarities between such models and humans, studies in animal models aid in our understanding of human diseases. The majority of laboratory studies on obesity use rats or mice [20]. Many researchers use mice as the primary model for obesity research. Variables such as age, different maturation cycles, and life-threatening diseases can affect the outcome of the experiments. As a result, these variables must be carefully considered during experimental design. The age range used in the majority of studies is between 4 and 18 weeks. Numerous studies on obesity employ female rats because the hormones involved are associated with slower body growth and increased fat storage in females [23,24]. Females have higher levels of hormones associated with metabolic changes, such as leptin [25] and adiponectin, which are implicated in food and energy intake. Numerous studies employ a high-fat diet (HFD) to induce obesity in rats [18,19,20,21,22,23], using fat sources such as beef tallow, lard oil, and soybean oil, but the preparations used vary in terms of lipid concentrations. The generally effective and easiest method for inducing obesity in animals is by exposing the animals to saturated HFDs containing fat as low as 13% of total energy (which is more than the 5% requirement of fat in rats) to as high as 85% of energy within 3–6 weeks. This diet ultimately affects the respective intracellular signaling pathways in hypothalamic target neurons with resulting changes in neuropeptide expression, by exhibiting reductions in insulin and leptin sensitivity, possibly in other brain areas of diet-induced obese rats [26,27].

To overcome the problem of obesity, several synthetic drugs have been introduced into the modern system of medicine. However, apart from being effective, most of these medications cause adverse side effects. Moreover, these drugs are not suitable for long-term use, as obesity requires long-term treatment, so choosing lipid-lowering substances that do not have negative side effects is important. The majority of approved anti-obesity medications, including rimonabant, amphetamine, and sibutramine, have been withdrawn due to an elevated risk of psychotic illnesses and nonfatal myocardial infarction or stroke [28]. Orlistat is currently the only therapy option for obesity due to its low risk of cardiovascular events [28,29,30]. Orlistat (Xenical) is a pharmacological agent that promotes weight loss in obese subjects by inhibiting gastric and pancreatic lipase, an enzyme necessary for the digestion of long-chain triglycerides [28,30]. At a three-daily dose of 120 mg, orlistat reduces fat absorption by 30% and is effective for both weight loss and maintenance [28,30].

Natural products and their active principles as sources for new drug discoveries and treatments of diseases have attracted attention in recent years. Herbs and spices are generally considered safe and have been proven to be effective against various human ailments. There has been a long history of the search for substances with the pharmacological potential to manage obesity, of which plants have been a huge source [31,32,33,34,35]. Solanaceae have been greatly employed among the plants utilized based on their history as medicinal and nutraceutical plants [36,37]. *Solanum melongena* (aubergine) and *Solanum aethiopicum* (Ethiopian eggplant) are two species of *Solanum* widely cultivated in Nigeria and across the African continent [38,39,40,41,42]. The fruit of *S. melongena*, commonly known as “Garden egg,” is an edible fruit or vegetable consumed worldwide. It is a rich source of free reducing sugars, anthocyanin, phenols, saponins, flavonoids, tannins, ascorbic acid, glycoalkaloids, and amide proteins [38,40,41,43]. Some medicinal properties have been ascribed to *S. melongena* fruits which are believed to be good for diabetic and obese individuals. For instance, several researchers have reported significant analgesic, anti-inflammatory, anti-asthmatic, anti-glaucoma, hypoglycemic, hypolipidemic, and weight reduction effects of eggplants, particularly on test animals and humans. [39,42,44,45,46]. In this study, we determined the regulatory potential of *S. melongena* and *S. aethiopicum* fruit-based diets on FTO gene expression levels in the hypothalamus, WATs, and BATs of experimental HFD-induced obese animals. Histopathological studies on the effects of *S. melongena* and *S. aethiopicum* on the WATs, hypothalamus, and liver of the experimental animals were also investigated.

## 2 Materials and methods

### 2.1 Plant materials

Fruits of *Solanum melongena* and *Solanum aethiopicum* were harvested from a farm community in Esa Anua, Uyo, Akwa Ibom State, Nigeria. The plants were identified and authenticated at the Bioresources Development and Conservation Programme Research Centre, Nsukka, Enugu State, Nigeria. These fruits were plucked and sorted by removing extraneous materials. The samples were air-dried, and the dried samples were pulverized and packaged in air-tight polyethylene bags.

### 2.2 Experimental animals

Sixty-nine (69) young female Wistar rats, weighing between 80 and 100 g at 6 weeks of age, were obtained from the animal house of the Faculty of Biological Sciences, University of Nigeria, Nsukka. The rats were housed in steel-topped aluminum partitioned cages at room temperature on a 12 h dark–light cycle and acclimatized to laboratory conditions for 14 days with *ad libitum* access to standard chow and water. To prevent food loss and contamination, diets were given to rats in special non-scattering cups. The rats were allowed access to water using glass tubes projecting through wire cages from inverted bottles inclined on one side of the cage.


**Ethical approval:** The research related to animal use has complied with all the relevant national regulations and institutional policies for the care and use of animals, and approval was granted by the Faculty Ethics and Biosafety committee, Faculty of Biological Sciences, University of Nigeria, Nsukka (UNN/FBS/EC/1019).

### 2.3 *Solanum* fruit preparation

Samples of *Solanum melongena* (SMF) and *Solanum aethiopicum* fruits (SAF) were air-dried, pulverized, and passed through a sieve (about 0.5 mm pore size) to obtain a fine dry powder. The SMF and SAF samples were stored in the refrigerator until further use.

### 2.4 Acute toxicity and lethality testing

The median lethal dose (LD_50_) of SMF and SAF was determined in experimental animals using the Lorke method [47] to define the range of lethal dose and safe dose of supplementation. For this testing, a total of 18 Swiss albino mice (weight range: 18– 28 g) were used. This method was carried out in two phases: phase 1 and phase 2. In the first phase, the mice were grouped into three and were treated orally via cannula with 10, 100, and 1,000 mg/kg body weight, respectively. The animals were placed under observation for 24 h for lethality or any behavioral changes. Based on the results of the observations, higher doses of 1,600, 2,900, and 5,000 mg/kg body weight were administered orally to another three groups of three mice each. They were placed under observation for 24 h to check for any behavioral changes or mortality. The LD_50_ was calculated using the formula:

{\text{LD}}_{50}=\surd ({D}_{0}\text{ }\times \text{ }{D}_{100}),]

where *D*_0_ is the highest dose that gave no mortality and *D*_100_ is the lowest dose that produced mortality.

### 2.5 Induction of obesity

The rats were weighed, grouped, and fed with water and a HFD for 6 weeks to induce obesity. The fat used in this study was animal fat and was gotten from beef tallow. A HFD was formulated by the modified method of Picchi (Tables 1 and 2) [48]. The composition of the feed was follows: carbohydrate: 250 g/kg; protein: 200 g/kg; fat: 500 g/kg; fiber: 20 g/kg; and vitamin–mineral mix: 30 g/kg. The composition of the feed was as follows: carbohydrate, 250 g/kg; protein, 200 g/kg; fat, 500 g/kg; fiber, 20 g/kg; and vitamin–mineral mix, 30 g/kg.

**Table 1:** Composition of a HFD for the induction of obesity in experimental animals

Nutrients	Source	Quantity (g/kg)
Carbohydrate	Maize	200
Protein	Processed soybean powder	250
Lipids	Beef tallow	500
Fibers	Husk/chaff from cereals	40
Vitamin–mineral mix	Commercially procured	10
Energy-value (kcal/kg)	—	6,460

Modified Picchi et al. [48].

**Table 2:** Proximate composition of the diet used in the study

Nutrients	Normal rat chow (%)	High-fat diet (%)
Moisture	8.2	5.6
Dry matter	92.5	94.4
Crude protein	35.71	21.80
Fat	12.34	46.03
Crude fiber	3.2	4.87
Carbohydrate	33.01	9.6
Ash	8.5	12.09

### 2.6 Experimental design

After establishing obesity in animals in groups 2–10, the rats in groups 4–10 were fed with specific rat diets having different amounts of SMF and SAF samples. The rest of the groups were fed with diets as described below, and the feeding was for another 6 weeks. The body weight changes and feed intake in the experimental groups were measured every 2 weeks and compared with the control group.

**Table:** 

Group 1: (Normal control)	Rats fed with a normal rat diet and water
Group 2: (Untreated control)	Rats fed with HFD and water
Group 3: (Standard control)	Rats fed with a normal diet and 12 mg/kgb.w of orlistat and water
Group 4: (SMF 10%)	Rats fed with SMF supplementation and water
Group 5: (SMF 20%)	Rats fed with SMF supplementation and water
Group 6: (SMF 30%)	Rats fed with SMF supplementation and water
Group 7: (SAF 10%)	Rats fed with SAF supplementation and water
Group 8: (SAF 20%)	Rats fed with SAF supplementation and water
Group 9: (SAF 30%)	Rats fed with SAF supplementation and water
Group 10: (15% SMF + 15% SAF)	Rats fed with SMF and SAF supplementation and water

### 2.7 RNA extraction and real-time polymerase chain reaction (RT-PCR)

Animals were sacrificed, and their adipose and hypothalamus tissues were stored immediately under liquid nitrogen after harvesting. Tissues were homogenized using a specific kit reagent suitable for lipid-rich tissues. Total RNA was extracted, and cDNA synthesis was performed using Qiagen RNeasy^®^ and *Accuprep* RNA extraction kits (Bioneer, Republic of Korea) following the manufacturer’s instructions. The sample was also treated with RNase-free DNase set for maximum purity, and the extracted RNA samples were quantified using a NanoDrop spectrophotometer. For cDNA strand synthesis, 1 μg of total RNA was reverse transcribed in a 20 μL final volume using random hexamers. Relative FTO gene quantitation was performed by thermal cycler using the Applied Biosystems Real Time-PCR RG 3000 (Corbette Research). The quality was assessed by running on a 2% agarose gel containing ethidium bromide. The bands were visualized using a UV lightbox (Figure S1). Primer sequences used for the FTO gene (Fwd 5′-TCCCAGAATTCCCCACTCAC-3′ Rev 5′-CTACCACACCGTCCTCATGT-3′) and GADPH (Fwd 5′-CTTGCCTCTCAGACAATGCC-3′ Rev 5′-AAGAAGATGCGGTCACCTCA-3′) are indicated and were designed using Primer 3 tool Software. Primer specificity was confirmed using BLAST searches, the appearance of a single band on gel electrophoresis, and melt curve analysis.

### 2.8 Histopathological study: slide examination and photomicrography

At the end of the study, sections of the brain, WAT, BAT, heart, liver, and kidneys were collected for histopathological examination. The tissue samples were fixed in 10% phosphate-buffered formalin for a minimum of 48 h. The tissues were subsequently trimmed, dehydrated in four grades of alcohol (70, 80, 90%, and absolute alcohol), cleared in three grades of xylene, and embedded in molten wax. On solidifying, the blocks were sectioned, 5 µm thick with a rotary microtome, floated in a water bath, and incubated at 60°C for 30 min. The 5 µm thick sectioned tissues were subsequently cleared in three grades of xylene and rehydrated in three grades of alcohol (90, 80, and 70%). The sections were then stained with hematoxylin for 15 min. Blueing was done with ammonium chloride. Differentiation was done with 1% acid alcohol before counterstaining with Eosin. Permanent mounts were made on degreased glass slides using a mountant: dibutylphthalate polystyrene xylene. The prepared slides were examined using a Motic™ compound light microscope using ×4, ×10 ×20, and ×40 objective lenses. The photomicrographs were taken using a Motic™ 9.0-megapixel microscope camera at ×400 magnifications; H&E ×400.

### 2.9 Statistical analysis

Relative mRNA expression was represented as a fold change over the calibrator sample (Figure S2). Comparative Ct (ΔΔCt) was calculated by subtracting ΔCt_(calibrator)_ from ΔCt_(treated samples)._ Relative fold changes were determined using the formula 2^−ΔΔCT^. The qRT-PCR data were done in replicates of at least three wells for the FTO gene, and one-way ANOVA was used to compare means of groups, followed by the Dunnette multiple comparison test. Data in the graph are presented as means ± S.E.M (*p < 0.05, **p < 0.01, ***p < 0.001, *p < 0.0001, and ns indicates *p* > 0.05). Graphpad prism version 8 was used for the analysis.

## 3 Results

### 3.1 SMF- and SAF-supplemented diets decreased feed consumption in high-fat diet-induced obese rats

To investigate the changes that occur following *Solanum* fruit supplementation, we first assessed the daily feed intake (g) of experimental animals from baseline to after 84 days (12 weeks) of treatment (Table 3). All measurements taken on day 0, right before starting the HFD, were considered baseline measurements. Throughout the study, Group 1 animals were allowed to eat regular rat chow. The remaining animal groups, namely groups 2–10, were fed a high-fat diet for 42 days in order to make them obese. The treatment period was divided into two parts: a 21-day post-induction phase and a 42-day post-induction phase, which are referred to as day 63 and day 84, respectively. The mean values of feed consumption for the control group (Group 1) grew progressively by 0.44 and 1.44 g during the two periods, day 63 and day 84, respectively. The increments for the untreated control (group 2) were 4.92 and 7.19 g/day, for days 63 and day 84, respectively. In comparison to the induction phase, the standard pharmacological treatment, orlistat, reduced the amount consumed by 2.69 and 3.69 g/day on days 63 and 84, respectively. Similarly, the daily diet consumption of SMF- and SAF-supplemented diet-treated rats was considerably lower than that of untreated control rats.

**Table 3:** Effect of SMF- and SAF-supplemented diets on food intake of HFD-induced obese rats

Groups	Baseline	Induction phase	Treatment phase	
	Day 0 (g)	Day 42 (g)	Day 63 (g)	Day 84 (g)
Control	12.33 ± 0.57^^a^^	14.89 ± 0.11^^b^^	15.33 ± 0.33^^b^^	16.33 ± 1.52^^c^^
Induced and untreated	12.33 ± 0.57^^a^^	10.02 ± 2.51^^a^^	15.00 ± 0.10^^b^^	17.21 ± 1.00^^c^^
Standard	12.33 ± 0.57^^a^^	10.02 ± 2.51^^a^^	7.33 ± 3.51^^a^^	6.33 ± 0.12^^a^^
SMF 10%	12.33 ± 0.57^^a^^	10.02 ± 2.51^^a^^	11.33 ± 4.93^^ab^^	12.30 ± 0.00^^b^^
SMF 20%	12.33 ± 0.57^^a^^	10.02 ± 2.51^^a^^	13.33 ± 1.54^^b^^	9.00 ± 0.00^^a^^
SMF 30%	12.33 ± 0.57^^a^^	10.02 ± 2.51^^a^^	13.66 ± 3.21^^b^^	6.06 ± 4.16^^a^^
SAF 10%	12.33 ± 0.57^^a^^	10.02 ± 2.51^^a^^	12.33 ± 2.51^^b^^	15.83 ± 0.22^^c^^
SAF 20%	12.33 ± 0.57^^a^^	10.02 ± 2.51^^a^^	10.00 ± 3.05^^a^^	11.11 ± 0.32^^b^^
SAF 30%	12.33 ± 0.57^^a^^	10.02 ± 2.51^^a^^	8.66 ± 2.51^^a^^	7.03 ± 0.00^^a^^
SMF 15% + SAF 15%	12.33 ± 0.57^a^	10.02 ± 2.51^^a^^	9.66 ± 0.57^^b^^	5.16 ± 0.31^^a^^

The data are presented as mean ± SD (*n* = 3). Statistical analysis for data comparison using ANOVA was done by comparing the groups against the control (group 1). Values with the same “letter(s) superscript” are not significantly different (*p* > 0.05), while values with different “letter(s) superscripts” are significantly different (*p* < 0.05).

### 3.2 The SMF- and SAF-supplemented diets decreased weight gain in rats fed a high-fat diet

After 6 weeks of induction, the rats fed a HFD showed significant obesity-related weight gain when compared to the normal diet group (control; Table 4). On average, the rats in group 2 gained weight throughout the experimental period, while significant decreases were observed in the treated groups, with the group fed a diet supplemented with SAF and SMF in combination showing the lowest weight gain.

**Table 4:** Effect of SMF- and SAF-supplemented diets on body weight (g) of HFD-induced obese rats

Groups	Baseline	Induction phase	Treatment phase	
	Day 0 (g)	Day 42 (g)	Day 63 (g)	Day 84 (g)
Control	100.21 ± 0.11^a^	135.00 ± 11.26^a^	150.33 ± 10.06^a^	165.33 ± 3.51^a^
Induced and untreated	100.21 ± 0.11^a^	222.33 ± 12.50^b^	218.00 ± 20.22^c^	215.33 ± 12.50^c^
Standard	100.21 ± 0.11^a^	222.33 ± 12.50^b^	188.66 ± 40.66^b^	143.33 ± 45.72^a^
SMF 10%	100.21 ± 0.11^a^	222.33 ± 12.50^b^	191.33 ± 10.96^bc^	185.33 ± 6.35^b^
SMF 20%	100.21 ± 0.11^a^	222.33 ± 12.50^b^	211.03 ± 18.91^c^	172.66 ± 5.13^ab^
SMF 30%	100.21 ± 0.11^a^	222.33 ± 12.50^b^	200.18 ± 7.37^c^	150.33 ± 17.89^a^
SAF 10%	100.21 ± 0.11^a^	222.33 ± 12.50^b^	212.66 ± 7.17^c^	209.00 ± 10.00^c^
SAF 20%	100.21 ± 0.11^a^	222.33 ± 12.50^b^	200.20 ± 11.55^c^	181.00 ± 50.86^b^
SAF 30%	100.21 ± 0.11^a^	222.33 ± 12.50^b^	183.00 ± 8.50^a^	161.33 ± 0.10^a^
SMF 15% + SAF 15%	100.21 ± 0.11^a^	222.33 ± 12.50^b^	172.00 ± 05.00^ab^	135.33 ± 12.50^a^

The data are presented as mean ± SD (*n* = 3). Statistical analysis for data comparison using ANOVA was done by comparing the groups against the control (group 1). Values with the same “letter(s) superscript” are not significantly different (*p* > 0.05), while values with different “letter(s) superscripts” are significantly different (*p* < 0.05).

### 3.3 SMF- and SAF-supplemented diets downregulated the relative FTO gene expression in the hypothalamus of high-fat diet-induced obese rats

**Figure 1:**
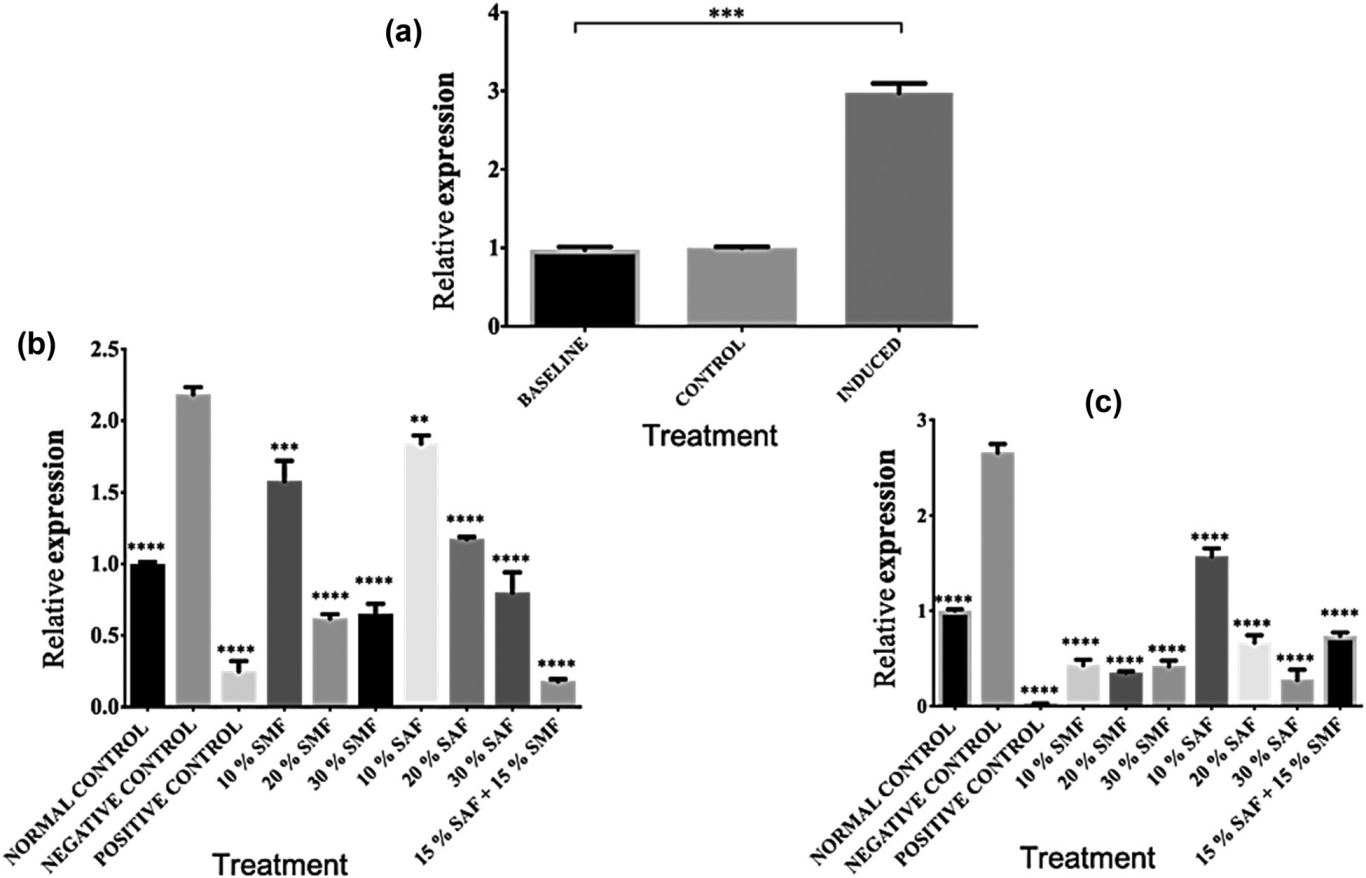
SMF and SAF treatments downregulated FTO gene expression in the hypothalamus. (a) Relative mRNA levels of FTO gene expression in the hypothalamus of baseline, control, and HFD-induced obese animals. (b) Three weeks of SMF and SAF supplementation significantly reduced FTO mRNA levels in the hypothalamus of HFD-induced obese rats. (c) Continued SMF and SAF supplementation to the 6th week further significantly downregulated the FTO mRNA levels in the hypothalamus of HFD-induced obese rats. Data in the graphs 1b, and 1c, are presented as means ± S.E.M compared to the untreated (negative) control (group 2) (*p < 0.05, **p < 0.01, ***p < 0.001, ****p < 0.0001, and ns indicates *p* > 0.05). All data depicted in the graphs represent three biological repeats.

The hypothalamus helps execute critical processes that regulate food intake, blood glucose levels, and energy circulation [49,50,51]. Disrupting this process has been associated with metabolic syndrome [52], of which obesity is a common cause. Our results showed that the consumption of HFD for six (6) weeks upregulated the FTO mRNA levels in the hypothalamus of all the animals (Figure 1a). As shown in Figure 1b, treatment with orlistat, SMF, SAF, or SMF + SAF for 3 weeks after induction of obesity significantly downregulated the mRNA levels of the FTO gene in all treatment groups in a dose-dependent manner when compared with the untreated control (group 2). At 6 weeks of treatment with orlistat, SMF, SAF, or SMF + SAF, the FTO expression was further downregulated for all the treatments when compared to the untreated group (Figure 1c).

### 3.4 FTO gene expression in the WAT of high-fat diet-induced obese rats was downregulated by SMF- and SAF-supplemented diets

**Figure 2:**
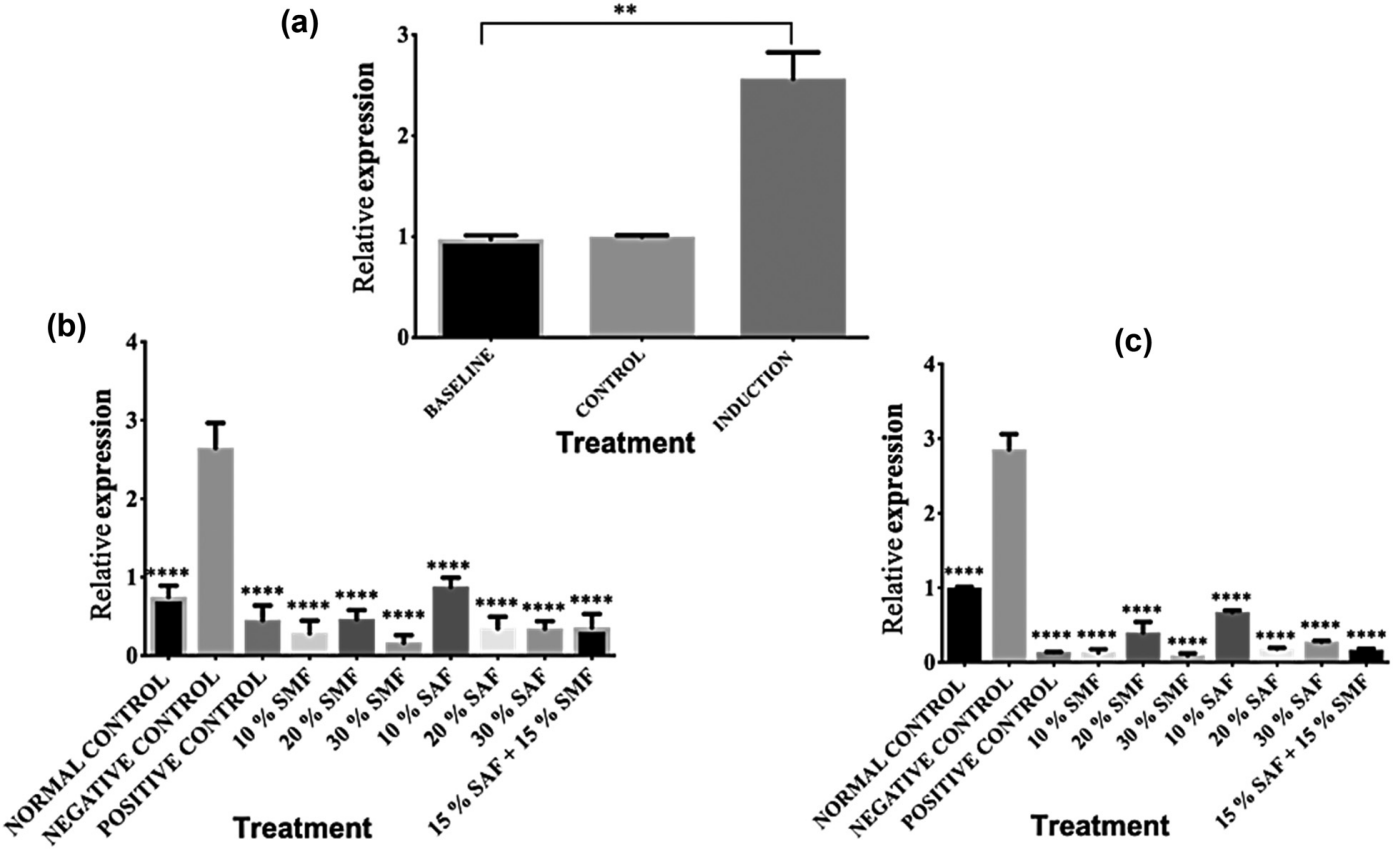
SMF and SAF treatments downregulated FTO gene expression in the WAT: (a) Relative mRNA levels of FTO gene expression in the WAT of baseline, control, and HFD-induced obese animals after 6 weeks. (b) Three weeks of SMF and SAF supplementation significantly reduced FTO mRNA levels in the WAT of HFD-induced obese rats. (c) Continued SMF and SAF supplementation to the 6th week further significantly downregulated the FTO mRNA levels in the WAT of HFD-induced obese rats. Data in the graphs 2b and 2c, are presented as means ± S.E.M compared to the untreated group (*p < 0.05, **p < 0.01, ***p < 0.001, ****p < 0.0001, and ns indicates *p* > 0.05). All the data depicted in the graphs represent three biological repeats.

The white adipocytes primarily make up the WAT, and its major role is in the storage of energy in the form of triglycerides. Obesity-related alterations frequently cause shape dysmorphology in WAT cells and other surrounding blood vessels, which may have long-term effects on body metabolism and associated disease conditions [53]. The mRNA expression of the FTO gene in the WAT of rats fed normal chow versus a HFD for 6 weeks to induce obesity is shown in Figure 2a. The data indicate that compared to the baseline and control, the consumption of HFD upregulated the levels of the FTO mRNA in the WAT of all the animals. Treatment with orlistat, SMF, SAF, or SMF + SAF for 3 weeks after induction of obesity significantly (****p < 0.0001) downregulated the mRNA levels of the FTO gene in all treatment groups in a dose-dependent manner when compared with the untreated group (Figure 2b). It was even more downregulated for all the treatments after 6 weeks of treatment with orlistat, SMF, SAF, or SMF + SAF than for the group that did not get any of them, as shown in Figure 2c.

### 3.5 The relative FTO mRNA level in the BAT of high-fat diet-induced obese rats was lowered by SMF and SAF-supplemented diets

**Figure 3:**
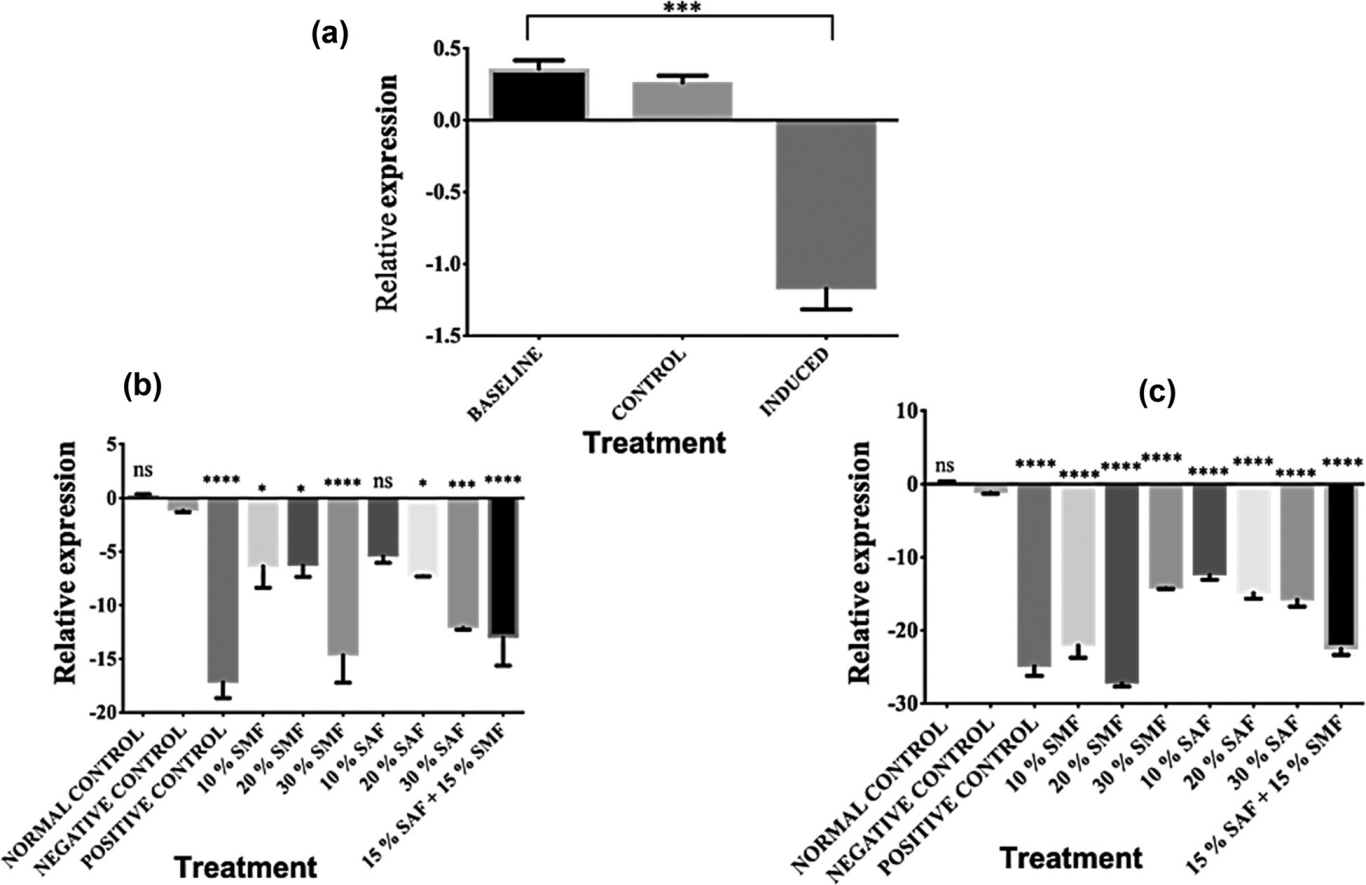
SMF or SAF treatment downregulated FTO gene expression in the BAT. (a) Relative mRNA levels of FTO gene expression in the WAT of baseline, control, and HFD-induced obese animals after 6 weeks of induction. Surprisingly, the consumption of HFD during the induction phase downregulated the expression of the FTO gene; (b) 3 weeks of SMF, SAF, or SMF + SAF supplementation significantly reduced FTO mRNA levels in the BAT of HFD-induced obese rats; (c) continued SMF and SAF supplementation to the 6th week further significantly down-regulated the FTO mRNA levels in the BAT of HFD-induced obese rats. Data in the graphs 3b, and 3c, are presented as means ± S.E.M compared to the untreated group 2 (*p < 0.05, **p < 0.01, ***p < 0.001, ****p < 0.0001, and ns indicates *p* > 0.05). All the data depicted in the graphs represent three biological repeats.

BAT is critical to body temperature regulation. It is predominantly made up of brown adipocytes that transform food energy into heat in a process called thermogenesis. A defect in this tissue has been linked to obesity and other closely related disorders such as chronic unresolved inflammation [54,55]. The mRNA levels of the FTO gene in the BAT of rats fed normal chow versus a HFD for 6 weeks are shown in Figure 3a. Surprisingly, the data showed that the consumption of HFD during the induction phase down-regulated the expression of the FTO gene. This is opposed to the observations in the hypothalamus and WAT. As shown in Figure 3b, treatment with orlistat, SMF, SAF, or SMF + SAF for 3 weeks after induction of obesity significantly down-regulated the mRNA levels of the FTO gene in all treatment groups (with the exception of group 7) in a dose-dependent manner when compared with the untreated group 2. Similarly, at 6 weeks of treatment with orlistat, SMF, SAF, or SMF + SAF, the FTO expression was further significantly (****p < 0.0001) downregulated for all the treatments when compared to the untreated group 2, as represented in Figure 3c.

### 3.6 Deleterious lesions in the WAT of high-fat diet-induced obese animals were ameliorated by treatment with *Solanum* fruit supplementation

**Figure 4:**
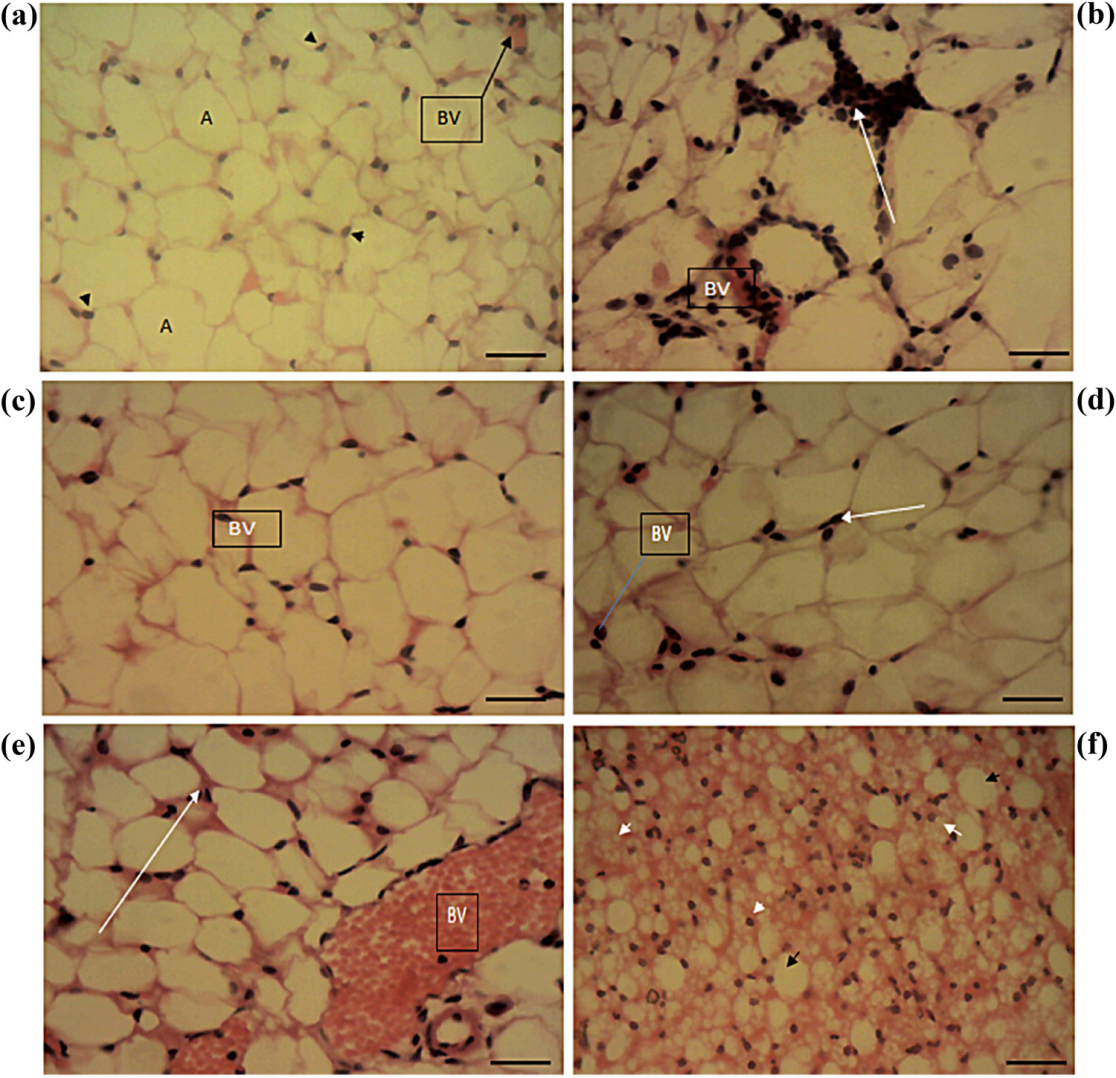
Sections of the WAT of the experimental animals: (a) WAT section of normal control (without obesity induction) showing normal histomorphology in experimental animals. (b) WAT of HFD-induced but untreated control, showing irregular reddish thickenings in adipocytes (white arrow), an increase in the size of adipocytes, and mononuclear leucocytic infiltrate droplets. (c) Adipocyte histomorphology and blood vessel (BV) histomorphology are normal in the standard drug-treated groups, but adipocyte size is relatively increased. (d) Rat fed with 30% SMF showing normal BV, normal lobules of blood vessels, and lipid cytoplasmic (white arrow), but with a relatively increased size of adipocytes. (e) The group fed with 30% SAF showed moderate fibrosis of the connective tissues, monomer infiltration (white arrow), and a slight increase in adipocytes. (f) Rat fed with 15% SAF + 15% SMF shows normal tissue histomorphology, normal BV, normal lobules of blood vessels, and lipid cytoplasmic droplet. However, it exhibits relatively smaller adipocytes and thickening of blood vessels. The photomicrographs were taken using a Motic™ 9.0-megapixel microscope camera at ×400 magnification. H & E, ×400. The scale bar represents 10 mm.

Normal control animals showed normal histomorphology in WAT, displaying normal adipocyte nuclei and blood vessels (Figure 4a). In the HFD-induced but untreated group, WAT showed changes consistent with obesity (Figure 4b), with a relative increase in the size and density of the adipocytes, irregular thickenings in adipocytes, and mononuclear leucocytic infiltrate droplets. After the 6-week treatment course, sections of WAT in the group with treatment using the standard drug (orlistat) showed normal histomorphology of the adipocyte and normal blood vessels but with a relatively increased size of adipocytes (Figure 4c). Animals fed with 30% SMF showed normal blood vessel lobules and cytoplasmic lipid (Figure 4d), whereas animals fed with 30% SAF showed moderate fibrosis of the connective tissues and infiltration of monomer and a slight increase in adipocytes (Figure 4e). Rats fed with 15% SAF + 15% SMF showed normal histomorphology, normal blood vessels, normal lobules of blood vessels, and lipid cytoplasmic droplets with relatively smaller adipocytes and reddish thickening of blood vessels (Figure 4f).

### 3.7 Treatment with *Solanum* fruit-supplemented diet abated hypothalamic lesions in high-fat diet-induced obese animals

**Figure 5:**
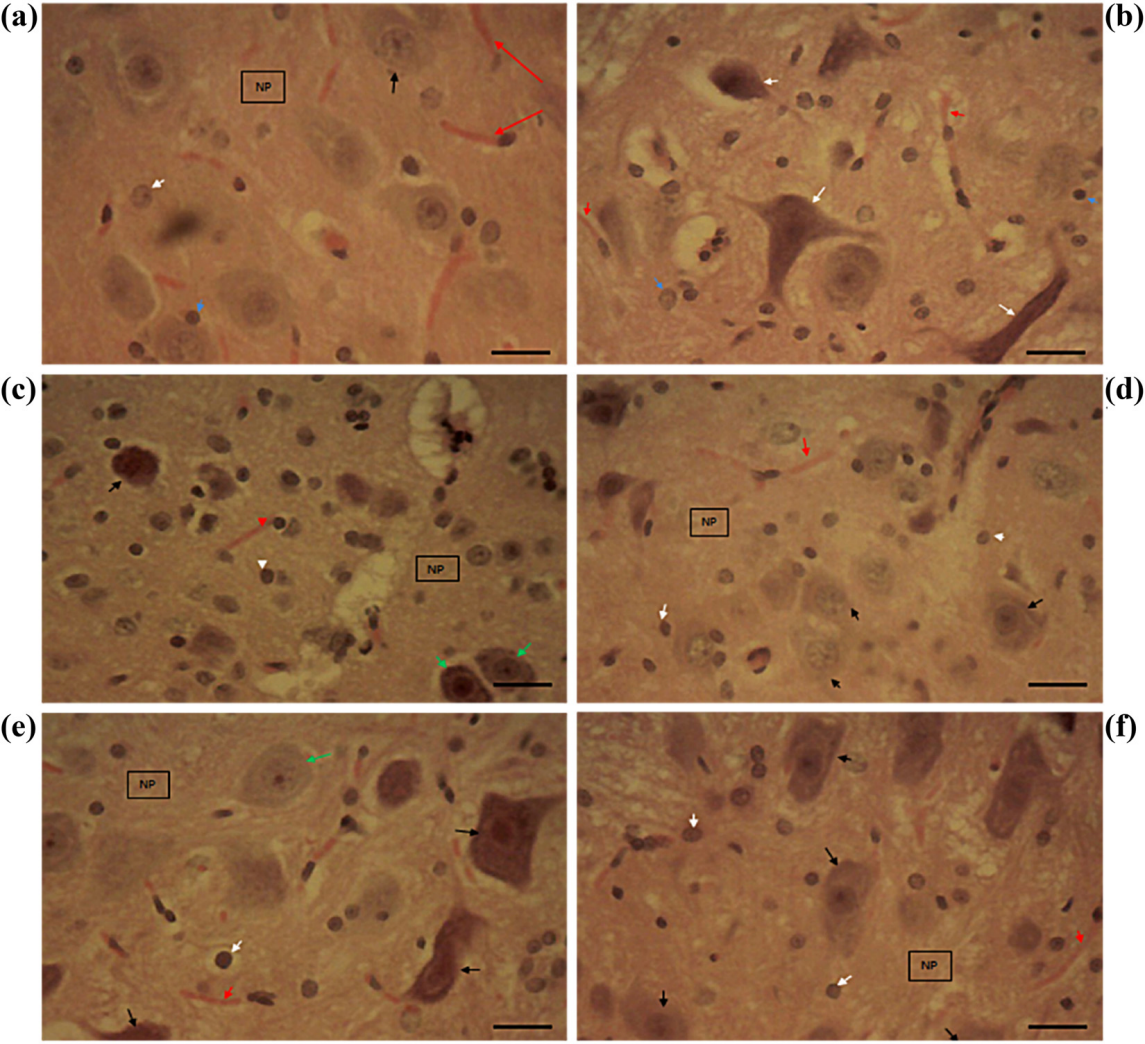
Micrographs of hypothalamic sections of the experimental animals: (a) Hypothalamic sections of the control group showing normal histomorphology. (b) Neuronal necrosis (white arrow), glial cells (blue arrow), capillaries (red arrow), and neuropil in induced but untreated animals. (c) Orlistat-treated group with mild necrosis in PVN and VN, degenerated neurons (black arrow) with nucleoli, mildly degenerated glial cells (white arrow), capillaries (red arrow), and neuropil (green arrow). (d) A 30% SMF-fed rat exhibits very mild necrosis degeneration in the PVN and VN. (e) No serious necrosis degeneration was observed in PVN or VN in rats fed 30% SAF. (f) Rats fed 15% SAF + 15% SMF showed no necrosis degeneration in PVN and VN. The photomicrographs were taken using a Motic™ 9.0-megapixel microscope camera at ×400 magnification. H & E, ×400. The scale bar represents 10 mm.

At the end of the study, the hypothalamus of control animals displayed normal neurons with prominent nucleoli, normal glial cells (astrocytes and oligodendrocytes), capillaries, and neuropils (Figure 5a). Sections of the induced but untreated group showed neuronal necrosis with deep basophilic cytoplasm alongside nuclei pyknosis and karyorrhexis, glial cells, capillaries, and neuropil (Figure 5b). The standard control group treated with orlistat showed mild degeneration of necrosis in the hypothalamic PVN and VN, mildly degenerated glial cells, capillaries, and neuropil (Figure 5c). Animals fed with 30% SMF showed very mild degeneration of necrosis in PVN and VN (Figure 5d). However, animals fed with 30% SAF showed no significant decrease in degeneration of necrosis in PVN and VN (Figure 5e). Likewise, animals fed with 15% SAF + 15% SMF showed no degeneration of necrosis in PVN and VN (Figure 5f).

### 3.8 HFD-induced obese animals fed with *Solanum*-supplemented feed displayed less severe liver lesions

**Figure 6:**
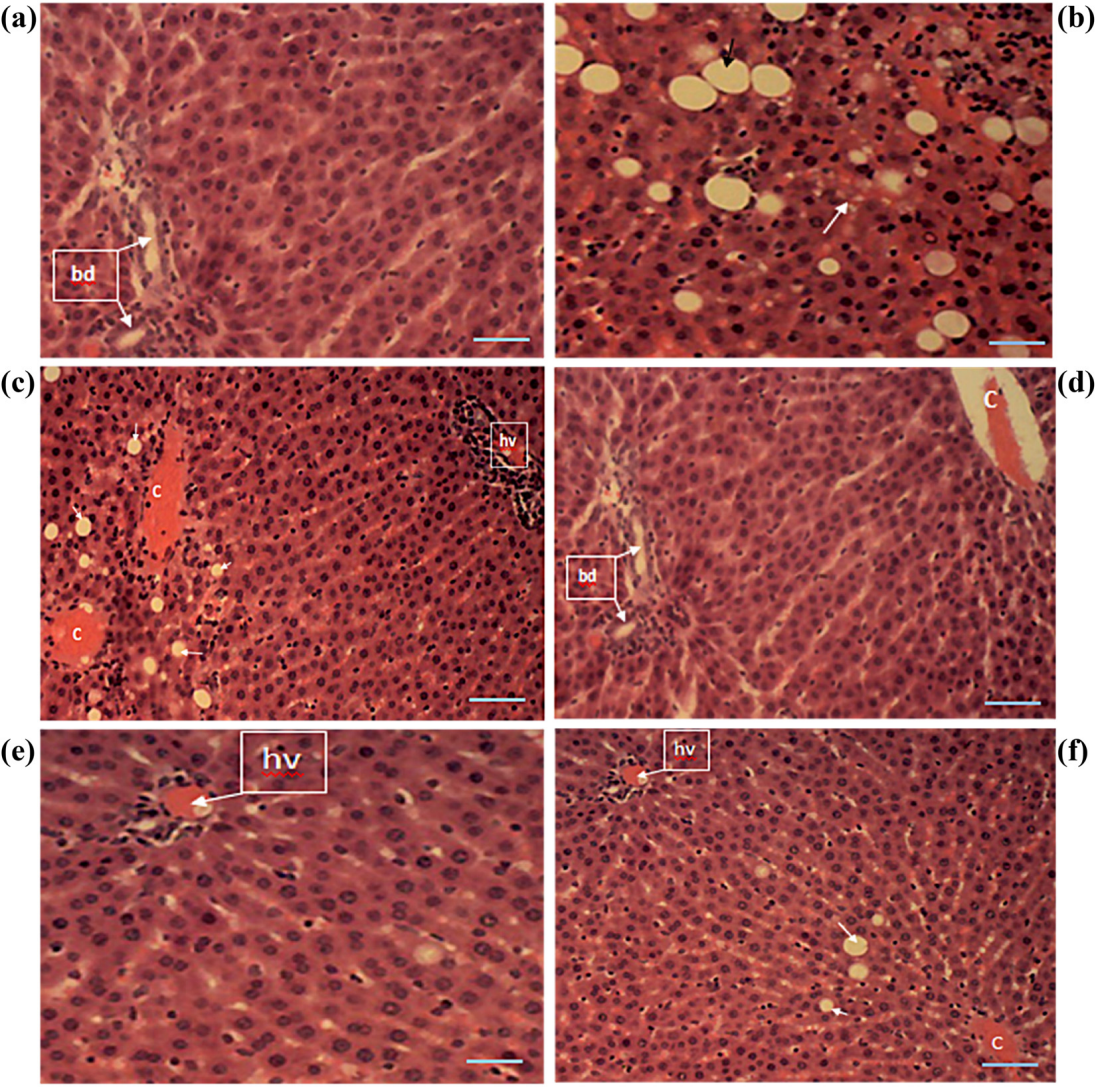
Micrographs of liver sections of experimental animals. (a) Liver sections of the control group showed normal histomorphology in experimental animals. (b) Liver sections of the induced but untreated group show histomorphological changes consistent with severe macrovesicular and microvesicular steatosis. (c) Liver sections of the standard-drug-treated group showed hepatocytes with multiple lipid vacuoles in their cytoplasms (white arrow). (d) Section of the liver of animals treated with SMF 30% showed normal histomorphology. (e) The group treated with 30% SAF showed mild microvesicular steatosis and intracytoplasmic lipid vacuoles. (f) Animals treated with SAF 15% + SMF 15% showed very mild microvesicular steatosis (white arrow) and large intracytoplasmic lipid vacuoles in the liver. The photomicrographs were taken using a Motic™ 9.0-megapixel microscope camera at ×400 magnification. H & E, ×400. The scale bar represents 10 mm.

After the complete treatment course, liver sections of control animals showed normal histomorphology with hepatocytes arranged as interconnecting cords around the central veins and normal portal triads, hepatic vein, hepatic artery, and bile ducts, and blood vessels (Figure 6a). Sections of the induced but untreated group showed histomorphological changes consistent with severe macrovesicular and microvesicular steatosis (Figure 6b). The affected liver cells showed a single large fat lobule in their cytoplasm, displacing and flattening the nuclei in an eccentric position. Sections of the standard-drug treated group showed hepatocytes with multiple lipid vacuoles in their cytoplasms (Figure 6c). Treatment with SMF at 30% showed normal histomorphology in experimental animals (Figure 6d). The group treated with 30% SAF showed mild microvesicular steatosis and intracytoplasmic lipid vacuoles (Figure 6g). Figure 6h shows the liver sections of animals that were given 15% SAF + 15% SMF. The animals had very mild microvesicular steatosis as well as large intracytoplasmic lipid vacuoles.

## 4 Discussion

Weight gain and obesity and the risk factors associated with these conditions have been on the increase in most parts of the world [1]. The detrimental effects of these conditions accentuate the need for effective preventive measures, hence this research. A diet containing a high percentage of lipids leads to the development of hyperlipidemia, atherosclerosis, abnormal lipid metabolism, and cardiovascular diseases, which are hallmarks of obesity [56,57]. Studies show that FTO rs8050136 allele (*n* = 36,973) was positively associated with the percentage of energy derived from fat and inversely associated with energy from carbohydrates in a cohort of 36,973 subjects [17]. FTO is a widely expressed gene in cells, and its transcriptional regulation is controlled by more than one factor, thereby forming a complex network [3,6,7]. Over-expression of this gene has been reported to lead to overweight and obesity [58,59]. In addition, FTO has also been shown to influence adipogenesis by regulating the process of mitotic clonal expansion in obese mice [60].

The use of medicinal and nutraceutical plants for the treatment and management of ailments has long been practiced due to their richness in various bioactive phytochemicals [61]. One such plant is the African eggplant, of which the fruits are commonly known as “garden eggs” and are largely consumed as edible foods. Eggplant has been shown to prevent and manage hypercholesterolemia and oxidative stress and can also modulate the expression of genes that may trigger obesity [62]. Our preliminary studies of the two varieties of garden eggs used in this study showed the presence of many phytochemicals, namely, alkaloids, flavonoids, saponins, tannins, glycosides, anthraquinones, anthocyanins, oxalate, phytate, hydrocyanic acid, haemagglutinin, and resins [40].

In this study, we set out to investigate the expression profile of the FTO gene in HFD-induced obese animals treated with supplementation of the two species of garden egg studied. First, we investigated the feed intake and weight reduction potentials of the feed supplemented with fruits of *Solanum* where we found a reduction in both indices. The dietary fiber content of the fruit samples may have played a role in the reductions in feed intake observed in the fruit-supplemented diet groups. Consumption of dietary fibers has been shown to increase feelings of satiety or fullness, which helps to prevent overeating [63,64]. In addition, the reduction in food consumption observed in the treated groups suggests that both fruit samples may have appetite suppressant potential, which may have contributed to the reduction in food consumption observed. This suggests that SMF and SAF are effective in the management of weight. Eggplants’ ability to aid in weight loss may be due to their low energy density, which can be attributed to the high moisture, high fiber, low carbohydrate, and low-fat content that they contain, among other factors. Additionally, the weight-reducing potential observed in both fruit samples could be attributed to the lipase inhibition potential of the fruits, which acts by inhibiting gastric and pancreatic lipase, which is an enzyme crucial for the digestion of long-chain triglycerides [65].

We then carried out further investigation by focusing on three main tissue types: the hypothalamus, the WAT, and the BAT. Studies have shown that the hypothalamus plays a critical role in the control of food intake, blood glucose levels, and energy circulation [49,50,51], and alterations in the hypothalamus have been linked to the development of metabolic syndrome [52]. The WAT, predominantly consisting of white adipocytes, functions in the storage of energy as triglycerides, and obesity-related WAT dysfunction is shown to inflict long-term consequences on body metabolism [53]. The BAT transforms food energy into heat, and defective thermogenesis is linked to obesity and closely related disorders such as chronic unresolved inflammation [54,55]. For WAT and hypothalamus, our results showed a proportionate increase in FTO expression following the induction of obesity, and treatment with SMF, SAF, or SMF + SAF consistently downregulated FTO gene expression in a dose-dependent manner when compared to the untreated group, with the 6-week treatment duration being more effective than the 3-week duration (Figures 1 and 2). The increase in the mRNA levels of the FTO gene in WAT following induction in this study is in line with previous reports that HFD led to a significant increase in the adipose tissue of induced obese mice. [3,66,67,68,69]. Likewise, a previous study reported that inactivation of the FTO gene in mice fed an HFD provoked a reduction in fat mass linked to obesity [70]. However, in contrast to our findings, a previous study reported that hypothalamic FTO mRNA is reduced upon induction of obesity by an HFD and that there was a trend for it to be upregulated in the fasted state, consistent with other previous reports of its upregulation during food deprivation in rats and mice [70,71,72,73]. These differences collectively may support the understanding that FTO transcription is regulated by energy status [74,75,76]. We investigated the response of the BAT to obesity induction with the same experimental conditions as for the hypothalamus and WAT. Surprisingly, contrary to the increase in the FTO mRNA observed in the hypothalamus and WAT, the expression of the FTO gene in BAT was downregulated (Figure 3a). This result is similar to the report that mice fed a HFD showed no physiological changes in the BAT [77], as they further explained that this effect could be attributed to the increase in thermogenesis in the BAT, which may be related to the suppressive effects of the dietary lipid on the fatty acid synthesis in this tissue through the help of uncoupling protein 1. We have not investigated the molecular mechanism of our observation as the scope of this study did not cover that. However, the decreases in mRNA levels of the induced groups could be attributed to the thermogenic potency of the BAT [78,79,80].

As we expected, treatment with SMF, SAF, or SMF + SAF for 3 weeks showed a further downregulation of the mRNA levels of the FTO gene in the BAT, except for 10% SAF (group 7) (Figure 3b). Continued treatment to the 6th week significantly downregulated the 10% SAF group while further downregulating the other groups (Figure 3c). In line with our results, previous studies on mice showed that treatment with eggplants reduced body weight and adiposity independent of the changes in food intake and also improved glucose tolerance [57]. The overall decrease observed in the FTO mRNA levels of rats fed *Solanum* fruit-supplemented feed in the hypothalamus, WAT, and BAT may be connected to the findings in our previous reports, which showed that both fruits possess bioactive compounds [40] with separate, combined, or synergistic therapeutic potentials. Studies have shown that one of the mechanisms by which fruits and vegetables prevent oxidative stress is the alteration in the expression of genes involved in the generation and removal of the reactive oxygen species [81]. For example, the peel of eggplant and its component flavonoids have been previously shown to modulate the expression of the antioxidant response element [82]. To put it into context, our previous reports on the *Solanum* fruits here studied showed a substantial amount of vitamins A (retinoic acid) and E content [40], and retinoic acid, which is the active form of vitamin A (the carboxylic acid form of vitamin A) has demonstrated health benefits related to adiposity [83,84]. Likewise, a study on mice showed that treatment with retinoic acid reduced body weight and adipose depot mass independent of the changes in food intake and improved glucose tolerance as well as insulin sensitivity, probably through adipokine expression [85,86]. Similarly, chronic dietary vitamin A supplementation (retinyl ester form) increases thermogenic potential in BAT and muscle, reduces body fat content, and partially inhibits the development of obesity in dietary and genetic models of mice and rats [85].

Next, at the end of the 6-week treatment period, we looked at the histomorphology of the WAT, hypothalamus, and liver. The control rats in the WAT had normal histomorphology (Figure 4a), with normal adipocyte nuclei and blood vessels [87,88,89,90,91]. The untreated group displayed adipocytes with irregularly thickened walls, exhibiting increased adipocyte size and mononuclear leucocytic infiltrates (Figure 4b) [92,93]. The standard drug effectively reduced the effects of obesity, as the sections showed normal adipocyte histomorphology and normal blood vessels (Figure 4c) [94,95,96,97], but with substantially larger adipocytes. The fruit supplementation clearly improved morphology when compared to the untreated group, as the 30% SMF-treated group showed normal blood vessel lobules and cytoplasmic lipid but with a relatively increased size of adipocytes (Figure 4d), the 30% SAF-treated group showed moderate fibrosis of the connective tissues, infiltration of monomer, and a slight increase in adipocytes (Figure 4e), and the 15% SAF + 15% SMF-treated group showed normal histomorphology [94,95,96,97]. It does, however, have smaller adipocytes and a thickening of the blood vessels.

The hypothalamus is essentially composed of three key components that regulate and control food intake: the ARC, the VN, and the PVN. However, ARC is responsible for energy balance and control of satiety and has been extensively studied in refs. [98,99,100,101,102]. Nevertheless, in this study, we tried to focus on the PVN and the VN, which have been shown to be activated in response to a HFD [103]. In the hypothalamic sections of control animals, normal neurons with prominent nucleoli, normal glial cells (astrocytes and oligodendrocytes), capillaries, and neuropils were seen (Figure 5a) [104,105]. On the other hand, the induced but untreated group had an unhealthy condition with neuronal necrosis, glial cells, capillaries, and neuropil (Figure 5b) [104,105]. Standard orlistat treatment resulted in only a slight deterioration of necrosis in the hypothalamic PVN and VN and glial cells, capillaries, and neuropil (Figure 5c). This suggests that orlistat may not completely alleviate the negative effects of obesity on the hypothalamus. The treatment with *Solanum* fruit demonstrated improvement, as the 30% SMF-treated group showed very moderate degeneration in PVN and VN nucleus (Figure 5f), while the 30% SAF-treated group showed no significant decrease in PVN and VN degeneration (Figure 5g). Similarly, animals fed 15% SAF + 15% SMF showed no necrotic degradation in PVN and VN (Figure 5h). Although we did not investigate the molecular mechanism behind this observation, a study like this one found that Ginkgo biloba extract improves obesity by stimulating the serotonergic system of the hypothalamus, which could be a possible explanation, pending further investigation [106].

The liver is at the center of metabolism, and the most adverse effect of unhealthy conditions often leaves traces of damage or abrasions on the liver tissues. Healthy hepatocytes were shown as normal interconnecting cords around the central veins, as well as normal portal triads and hepatic veins, normal bile ducts, and blood vessels in the liver micrograph of the control animals [107,108,109]. Based on previous research, it has been shown that HFD, which is the most common cause of hepatocellular necrosis production in the liver (owing to free radicals or oxidative damage), can produce a variety of additional abnormalities throughout the body [90]. On the other hand, the untreated group showed histomorphological changes consistent with severe macrovesicular and microvesicular steatosis, with the affected hepatocytes displaying a single large fat lobule in their cytoplasm (Figure 6b). They also show displaced and flattened nuclei in an eccentric position [108,109,110,111,112,113]. Even after treatment with orlistat, hepatocytes having numerous lipid vacuoles in their cytoplasms were still observed (Figure 6c). The treatment with SMF at 30% resulted in normal histomorphology in experimental animals (Figure 6d); similarly, the treatment with SAF at 30% resulted in mild microvesicular steatosis and intracytoplasmic lipid vacuoles (Figure 6e), and the treatment with 15% SAF + 15% SMF resulted in very mild microvesicular steatosis and large intracytoplasmic lipid vacuoles (Figure 6f). Our findings demonstrate that the fruit of *Solanum* has the ability to treat HFD-induced obesity by lowering the FTO gene expression level, as well as curative properties for obesity-related damage to adipose tissue, the hypothalamus, and the liver when taken simultaneously.

## 5 Conclusion

The study reported herein revealed that SMF and SAF possess anti-obesity properties via the modulation of mRNA levels of the FTO gene. The histopathological investigation further showed almost normal patterns following the treatment of obesity-induced animals with *Solanum* fruits. This implies that both fruits, in addition to their ability to ameliorate obesity factors, also have the ability to facilitate the healing of some of the HFD-induced histomorphological damage to the liver, hypothalamus, and adipose tissues. Hence, these fruits are highly recommended for direct consumption or as part of a formulation for the management of obesity.

## Supplementary Material

Supplementary Figure
