# Supplementary material for: FTO gene expression in diet-induced obesity is downregulated by Solanum fruit supplementation
Source: Open Life Sci. 2022 Jun 15;17(1):641–58. doi: 10.1515/biol-2022-0067 (PMC9202533; doi:10.1515/biol-2022-0067)
Supplement: Supplementary Figure [file biol-2022-0067-sm.pdf]

Supplementary material

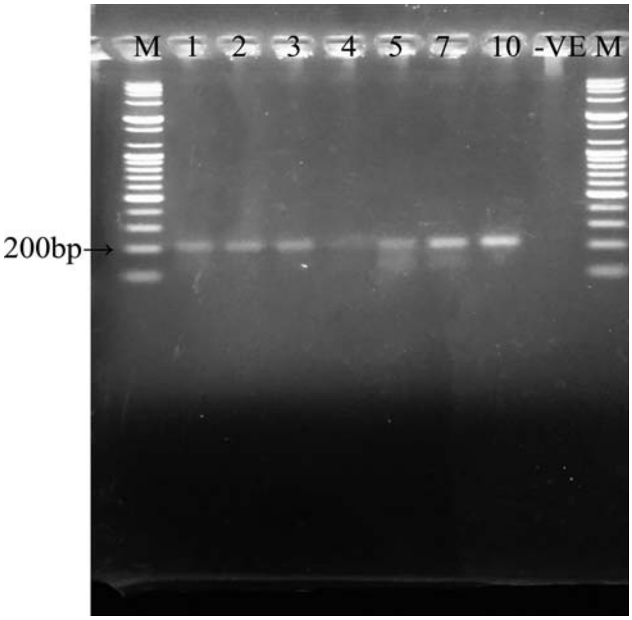

Figure S1: Gel image of PCR products of Amplified cDNA of FTO gene. Lanes 1, 2, 3, 4, 5, 7 and 10 represent the amplified cDNA region of the FTO gene. After amplification, the gel image of the PCR products showed sharp DNA bands corresponding to about 200 bp when compared to the DNA ladder. No band was observed in the negative control. M = DNA Molecular Weight Marker, –VE = Negative Control.

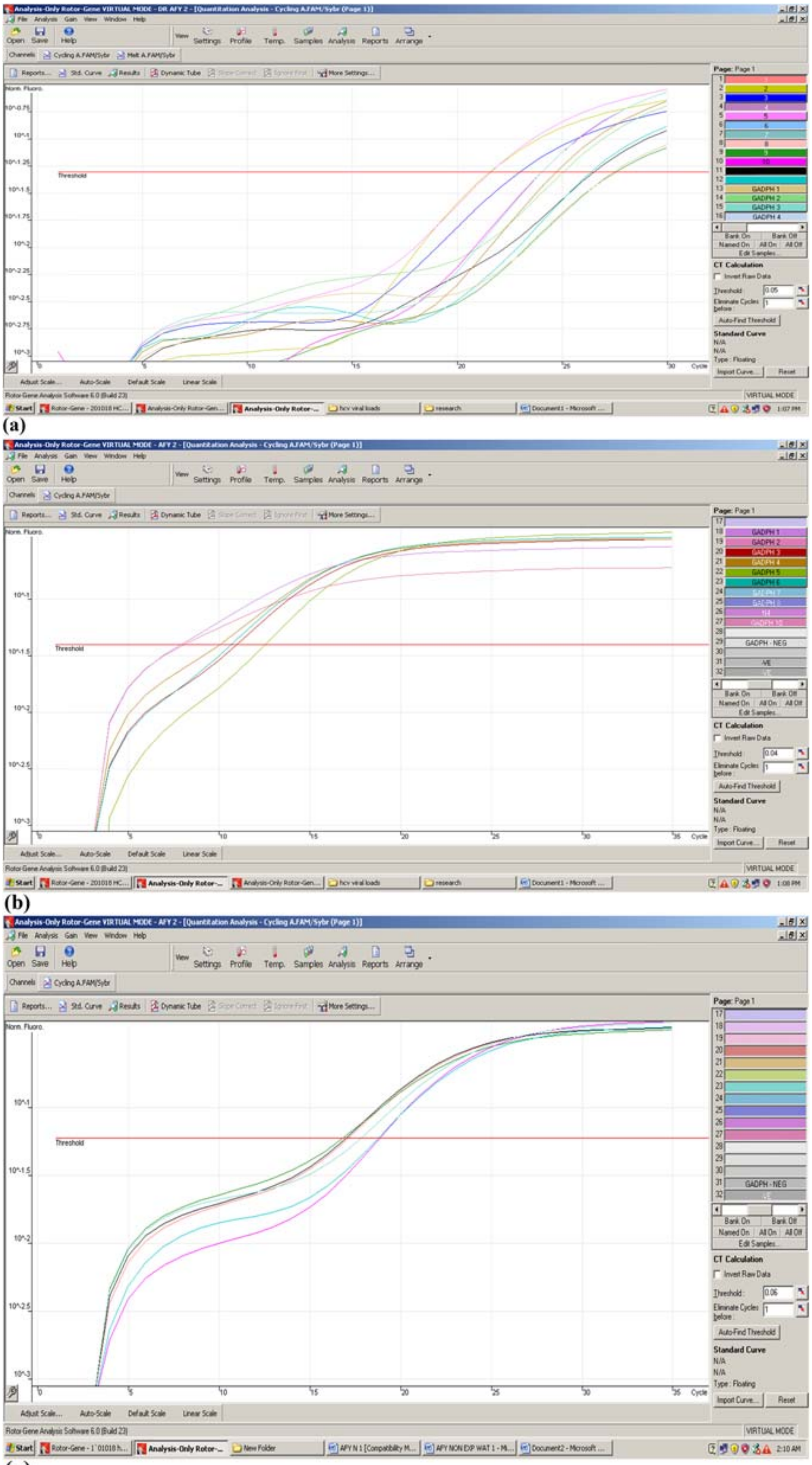

(a) (b) (c)

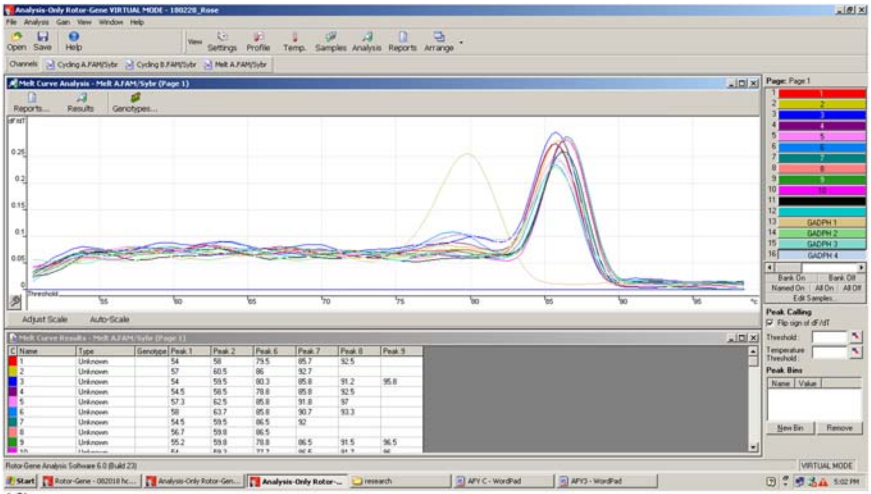

(d)

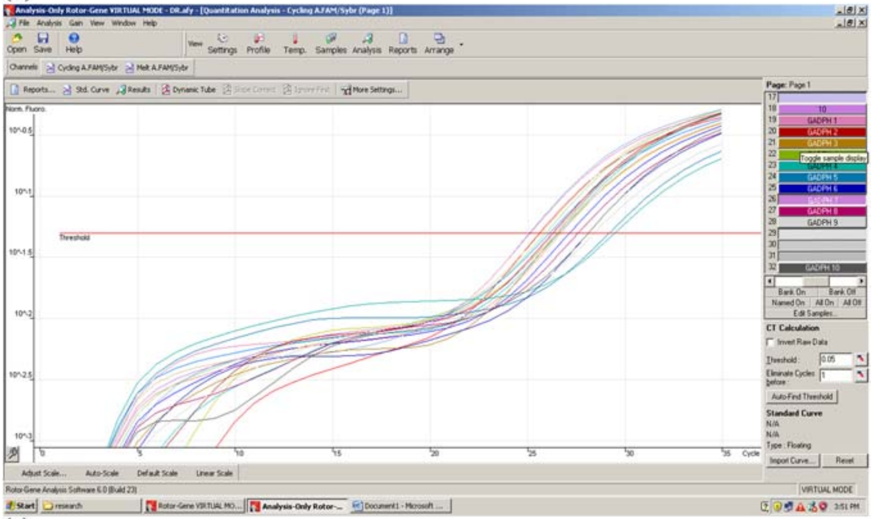

(e)

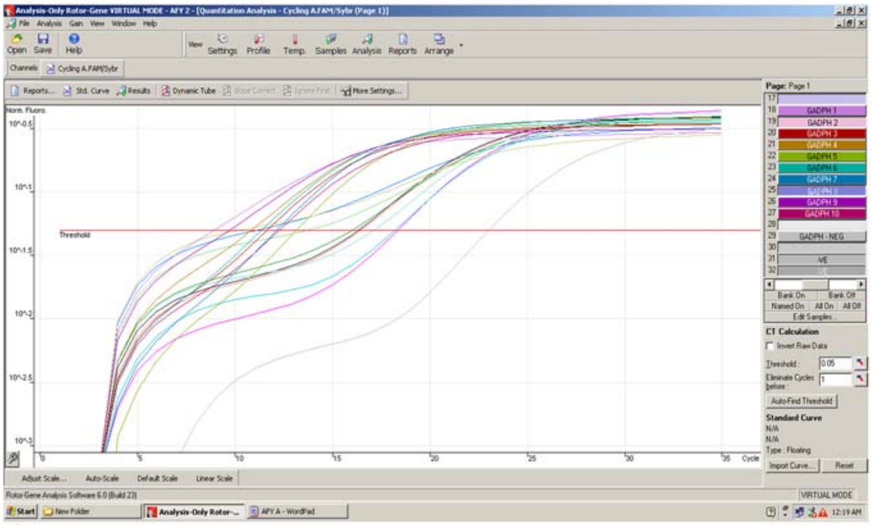

(f)

Figure S2: (a) Representative image of amplification curve for the brown adipose tissue (Day 63). (b) Representative image of amplification curve for the brown adipose tissue (BAT) day 84. (c) Representative image of amplification curve for the Hypothalamus (Induced Phase) (d) Representative image of amplification curve for the Hypothalamus (Induced Phase). (e) Representative image of amplification curve for the white adipose tissue, day 63. (f) Representative image of amplification curve for the white adipose tissue, day 84.
